# Do intense weather events influence dogs' and cats' behavior? Analysis of owner reported data in Italy

**DOI:** 10.3389/fvets.2022.973574

**Published:** 2022-09-02

**Authors:** Clara Palestrini, Giulietta Minozzi, Silvia Michela Mazzola, Annalaura Lopez, Simona Cannas

**Affiliations:** Department of Veterinary Medicine and Animal Science, University of Milan, Lodi, LO, Italy

**Keywords:** dog, cat, behavioral problems, owners, extreme weather events

## Abstract

Climate change is a threat to global health and can affect both veterinary and human health. Intense weather events, including sudden and violent thunderstorms or periods of extreme heat, are predicted to rise in frequency and severity and this could lead owners to significantly change their habits and schedules based on the weather, could modify human management and could aggravate pre-existing behavioral problems in pets. The aims of the present study were to identify and quantify possible weather events impact on management, behavior, and behavioral problems of Italian dogs and cats, based on previous owners' experiences with their animals. Two questionnaires were prepared, one for dogs and one for cats, investigating owners' perceptions of the impact of weather events on their pets' behavior. A number of 392 dogs and 426 cats' owners answered the questionnaire. Our study showed that many behaviors in both species were equally modified by environmental temperature. Play and activity increased with cold weather and decreased with heat, and sleep increased with drops in temperature and with hot weather. In particular, the increase in activity in correspondence with the thermic drop was more significant in males, while the increase in playing behavior was statistically greater in the Sheepdogs and Cattle dogs –group1. Weather events did not affect aggressive and house soiling behaviors in both dogs and cats, but weather events, including wild thunderstorms, torrential rains influenced the pets' behavior. Understanding how pets modify their behaviors based on a different owners' schedule and to weather events can help to refine prevention strategies through societal changes and owner education.

## Introduction

Around the world, companion animals are part of human societies (1, 2), and provide people with companionship, improved mental and physical health (including reduced depression, increased levels of oxytocin and decreased blood pressure and cholesterol levels), and expanded social networks. Many of these roles depend on physical activity, but it has been shown that also children and teenage development may benefit from living with pets (3–6). Many aspects of human society benefit from pets: simple companionship, but also work services, such as visual and hearing assistance dogs, medical detection dogs, and military working dogs (7, 8). Worldwide, the statistics describing the numbers of companion animals are scarce. However, according to Vetnosis and European Pet Food Industry Federation, in 2014, there were 223 million registered companion dogs and 220 million registered companion cats worldwide. Many owners consider their dogs and cats as family members and show great concern for their wellbeing. Those owners are prone to invest considerable resources for food and water requirements, living spaces, health conditions, and even pet's emotions and feelings (9–11). Over the years, co-evolution shaped the relationship between humans and pets, influencing human management and pets' behavior. Intense weather events could affect both factors, modifying human management and aggravating pre-existing behavioral problems, since owners could significantly change their habits and schedules based on the weather.

Global climate change is a One Health crisis, threatening both animal and human health (12, 13). Climate change is listed among the World Health Organization's top ten threats to Global Health in 2019, with heat-related illness predicted to contribute to an additional 250,000 human deaths annually by 2030 (14). All organisms live within a limited range of body temperatures, and temperature extremes result in functional constraints. Thermal windows evolved to be as narrow as possible to minimize maintenance costs, resulting in functional differences, between species and subspecies in various climate zones and between populations of a species (15). Direct effects of climatic warming are related to decrements in the organism's performance in growth, reproduction, foraging, immune competence, competitiveness, and behaviors. Moreover, performance in animals falls below its optimum during cooling and warming (15). Since extreme weather events, including thunderstorms, torrential rains, and flooding, are predicted to rise in frequency and severity (16), increased attention is necessary to identify and implement adaptation strategies (17). During last years, changes in temperatures and precipitation patterns have been evidenced in Italy, with events of extreme heat, torrential rains lasting for days and sudden and violent thunderstorms increasingly frequent (18), likely leading owners to change their routine based on the weather conditions. Unfortunately, in these circumstances, pre-existing pathological conditions, such as pathological anxiety or fear, thunderstorm phobia, and cognitive dysfunction in older dogs could worsen (19). Changes in daily routines can create a condition of distress with consequent modification of the main behavioral patterns (20), worsening all forms of anxiety and exacerbating problems such as urinary marking (21, 22), separation anxiety (23) and cognitive dysfunction in elderly subjects (24). Furthermore, subjects who previously suffered from fears or phobias toward loud noises can worsen the symptoms during sudden and violent thunderstorms (25).

At present, there is no specific literature on the subject. To the authors' knowledge, only one study in English (26) has examined the potential environmental impacts related to a dog's entire life cycle. Few studies investigated the most common triggers of heat-related illness and injuries in dogs in the UK (7, 17, 27) and the environmental impacts of companion dog's and cat's food consumption (26). However, limited attention has been given to the environmental factors contributing to canine and feline behavioral changes.

Therefore, the present study aimed to identify and quantify the possible climate change impact on management, behavior, and behavioral problems in an Italian pet dog and cat population.

## Methods

### Data collection

Two questionnaires were created to obtain owner-reports of the impact of weather events on caretaking as well as their pets' behavioral changes and administered to owners online through social media channels, personal contacts, and information disseminated to veterinarians.

Participants were given written information about the aim and the procedures of the study and the right to withdraw. Written informed consent was obtained from each participant. Participation was voluntary. No sensitive data were collected in both questionnaires, and complete anonymity was guaranteed. The study was approved by the Ethics Committee (reference number 5021, date 18/05/2021). All methods were performed in accordance with relevant guidelines and regulations. To meet key requirements for enrolment the survey targeted Italian participants older than 18 years, primary caregivers of a least one dog or one cat.

The questionnaires included three sections (see Supplementary materials 1, 2). The first section contained demographic information on participants (age, gender, number of adults and children in the household), the dog/cat's characteristics and history, and the physical and social environment of the dog/cat. Questions inquired about the home environment (apartment vs. house, urban vs. rural), pets “management (strictly indoor or the number of walks outdoor, place to rest, daily routine), age (current, age at acquisition), sex, reproductive status (entire or neutered/spayed), breed, source of dog/cat (breeder, pet store, shelter, rescue, family, friends, or stray), and the number of dogs or cats in the household. The second section targeted questions on specific behavioral patterns (sleeping, feeding, drinking, activity level). The survey also investigated whether the weather events change modifies the owners” animal management and the animal behavioral patterns based on previous experiences with their dogs or cats, including the most common behavioral problems (such as aggression, urine marking, fear of loud noises, thunderstorm phobia, etc.) that could be influenced by intense weather events.

### Data analysis

Data were entered into Microsoft Excel (Microsoft Corporation, 2010, Washington, DC), before being analyzed with SPSS statistical package (SPSS Statistic 27, IBM, Armonk, NY). Descriptive statistics (frequencies and percentages) were calculated to provide a general description of the sample. The data were not normally distributed, so were analyzed using non-parametric test: Kruskal-Wallis and X square test was used to investigate possible associations between the characteristics (e.g., age, sex, and breed) and management of pets and changing in behavior during weather events. Statistical significance was accepted at *p* < 0.05. A multivariate statistical analysis, principal component analysis (PCA) was used to analyze behavioral traits based on i) increased temperature and ii) after thunderstorms to determine the role of variables and detect common features. PCA plot was used to evaluate the distribution of the subjects according to the considered variables and contribution of the behavioral traits in the two settings. PCA analysis was conducted with the “princomp” function included in the default “stats” package, in R statistical environment version 3.6.2 (www.r-project.org). All PCA graphical representations were produced using R (www.r-project.org).

## Results

We collected reports from 392 people who answered the dog questionnaire and 426 the cat questionnaire. In both cases, the highest percentage was represented by women (87% dogs and 87.3% cats) aged between 21 and 40 years (55.1% dogs and 49.7% cats) living in couple (dogs 44.4% and cats 46.7%) without children (80%). Most of the owners who answered the questionnaire lived in northern Italy (69.4% for dogs and 68.1% for cats) in an urban environment (65% dogs, 71.1% cats). Owners believed that weather events had an effect on their pet's behavior (65.3% dogs, 66.9% cats). In particular, the number of purebred cat owners who believe that weather events influenced their cats' behavior was statistically greater than that of European shorthair owners (*p* < 0.05).

### Dogs

FCI classification was used to identify dog breeds. The most represented dogs were crossbreeds (44.4%), followed by Sheepdogs and Cattle dogs (except Swiss Cattle dogs) - group 1-(13%) and Retrievers-Flushing Dogs-Water Dogs-group 8-(9.2%). All other breeds were represented in a smaller percentage. Most dogs were adopted between 2 and 4 months of age (69.6%) and, to a lesser extent, after 5 months (28.1%). Many came from a private individual (34.4%), although a fair number were adopted from kennels (27.3%) or breeders (27%). Dogs included in the study were almost equally distributed between males and females (spayed female 39.3%, intact male 32.9%, neutered male 14.3%, and intact female 13.5%), and were mainly between the ages of 3 and 7 (37.8%) and between 13 months and 3 years (25%). Senior dogs aged 8 to 10 years represented a smaller percentage (15.6%). Most dogs (54.3%) were the only pet in the house. A specific daily routine was followed by most of the dogs (87%) even if the number and duration of walks varied in relation to the outside temperature both in periods of extreme heat (number 79.1%, duration 87.5%), and extreme cold (number 66.1%, duration 76.4%). The walk for 71.7% of dogs lasted from 15 to 60 min and was repeated two to four times a day (79.8%).

Dogs' play behavior and activity were considered normal by 79.1% of owners. These behaviors increased with sudden temperature drops (activity 60.7%, play 56.6%). In particular, the increase in activity in correspondence with the thermic drop was more significant in males (*p* < 0.05), while the increase in playing behavior was statistically greater (*p* < 0.05) in the Sheepdogs and Cattle dogs –group1. Most dogs had their own place to sleep (98%), evidenced by having a personal bed (45.7%), although some of them (20.2%) preferred the owners' bed. Sleeping behavior, defined as normal by most owners (92.1%), increased both when the temperature dropped (58.2%) and when the air temperature was excessively hot (66.6%).

Eating behavior, defined normal by most owners (74.5%), increased when there was sudden thermic drops (63%) and decreased with hot temperature (58.7%). Owners reported a significant decrease in appetite due to heat in intact males (*p* < 0.05).

Most owners (68.9%) defined their dogs' grooming behavior as normal. According to owners, this behavior tended to increase (39.3%) in the warmer seasons slightly.

In our sample, 41.1% of dogs never showed aggressive behavior toward owners, while inter-dog aggression was reported in 33.2% of dogs. House soiling was never reported by 59.9% of the owners, although a fair number of dogs (40.1%) have had episodes of inappropriate elimination in the home. Climatic change didn't seem to have any effect on aggressive and house soiling behaviors.

Owners defined 58.4% of dogs as very reactive/nervous in response to environmental stimuli, but only 11% of owners believed that their dog barked excessively, while 25% believed that their dog's vocalizations were normal.

About half (49.7%) of the dogs considered in our sample were not afraid of loud noises, gunshots and thunderstorms, while a smaller percentage sought for owner attention (21.4%). In correspondence with heavy thunderstorms or high-intensity incessant rains, dogs showed an increase in nervousness and reactivity (45.9%), fearful behaviors (47.7%), tendency to hide (38.8%), and vocalizations (26.5%). There was also a reduction in activity (48%) and play behavior (33.7%).

Fear behaviors (14%) and vocalizations (7%) are significantly less present in dogs adopted from breeders (*p* < 0.05). Owners' and dogs' ages did not affect the behaviors considered in the questionnaire.

### Cats

European shorthair was the most represented in the questionnaire (85%), followed by smaller percentages of cats of different breeds. Most of the cats were adopted between 2 and 4 months of age (75.6%) and, to a lesser extent, after 5 months (18.8%). Many cats came from colonies -feral cats living outdoors in groups- (34.3%), and about the same number came from a private individuals (31.9%), although a fair number came from rescues (23.7%); cats adopted from breeders were a small percentage (8.5%).

Our sample of cats was almost equally distributed between males and females (spayed female 47.2%, spayed male 44.6%, intact female 4.5%, and intact male 3.8%), mainly between the ages of 3 and 7 (35.9%) and between 13 months and 3 years (26.3%). Senior cats (over 10 years) represented a smaller percentage (12.9%). Most cats (42.5%) were the only pet in the house, although as many (41.8%) lived with another cat.

Most of the cats followed a specific daily routine (78.4%). Cats mostly (63.4%) lived in apartments, and 53.3% were exclusively indoor, while 46.7% had access outdoor. Number and duration of outside activities varied in relation to the outside temperature, both in periods of extreme heat (number 65.1%, duration 75.3%) and extreme cold (number 69.5%, duration 75.8%).

Cats' play behavior and activity were considered normal by 70% of the owners. Owners reported that these behaviors changed during weather events. In particular, extreme heat led to a decrease in the level of activity (76.6%), while this behavior tends to increase with cold temperature (66.1%). Play behavior also increased in relation to temperature lowering (63.8%).

Most cats had their own place to rest (93.7%), and in 38.7% the preferred resting place was the owners' bed. Sleeping behavior, defined as normal by most owners (91.1%), increased both when the temperature dropped (76.4%) and when the climate was excessively hot (77.2%). Eating behavior, defined as normal by most owners (76.5%) increased with sudden thermic drops (78.7%) and decreased with hot temperature (71.8%). Food was always available for most of the cats (81%).

Most owners (87.1%) described grooming behavior as normal. This behavior tended to slightly increase (46.9%) in the colder seasons. In our sample, 37.1% of cats never showed aggressive behavior toward owners, while inter-cat aggression was reported in 25.3% of cats.

Inappropriate elimination was never reported by 58.7% of the owners, although a fair number of cats (41.3%) have had episodes of house soiling. Marking behavior by rubbing their face or scratching on objects was shown by 70.7% of the cats considered in our sample. Climatic change did not appear to have any effect on aggressive and house soiling behaviors.

64.8% of the owners defined their cats as very reactive/nervous in response to environmental stimuli, but only 15.3% of owners believed that their cat vocalized excessively, while 61.5% believed that their cat's vocalizations were normal.

Most (60%) of the cats considered in our sample were afraid of loud noises, gunshots, and thunderstorms, while a smaller percentage (31.9%) did not show any behavioral reaction toward these stimuli. In correspondence with heavy thunderstorms or high-intensity incessant rains, cats showed an increase in nervousness and reactivity (46.8%), fearful behaviors (57.7%), tendency to hide (55.6%), and vocalizations (30.8%). Fear behaviors were significantly higher in cats older than 1 year of age and adopted from rescues (*p* < 0.05).

The age of the owners and the age of the cats did not affect the behaviors considered in the questionnaire.

### PCA analysis

PCA results are shown using the first two components (PC1 and PC2) as axis (Figures 1–8). These two components together explained 48.2% of the variation for dogs and 49.2% for cats with the increase in temperature, and 65.5% of the variation for dogs and 62.5% for cats with the increase in thunderstorm.

**Figure 1:**
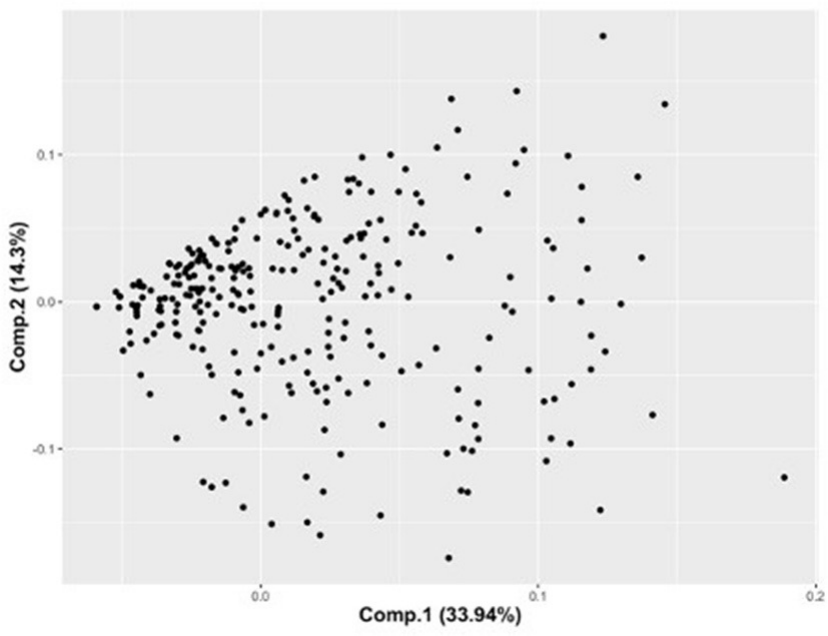
Score plot of dogs on the first (PC 1) and second (PC 2) principal components, with the increase in temperature.

**Figure 2:**
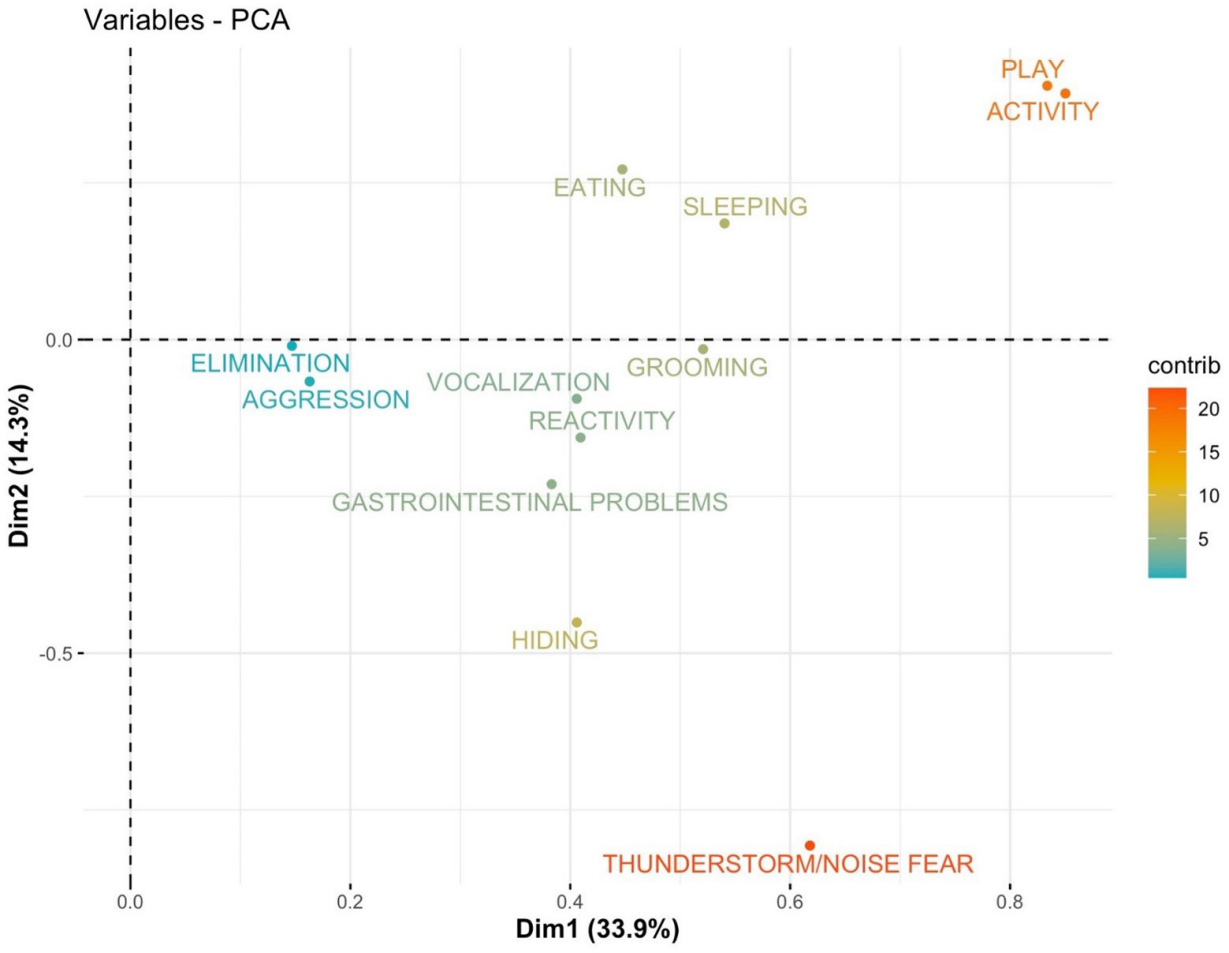
Loadings plot of the behavioral variables of dogs on the first (PC 1) and second (PC 2) principal components with the increase in temperature. The contribution of the variables is colored depending on their importance.

**Figure 3:**
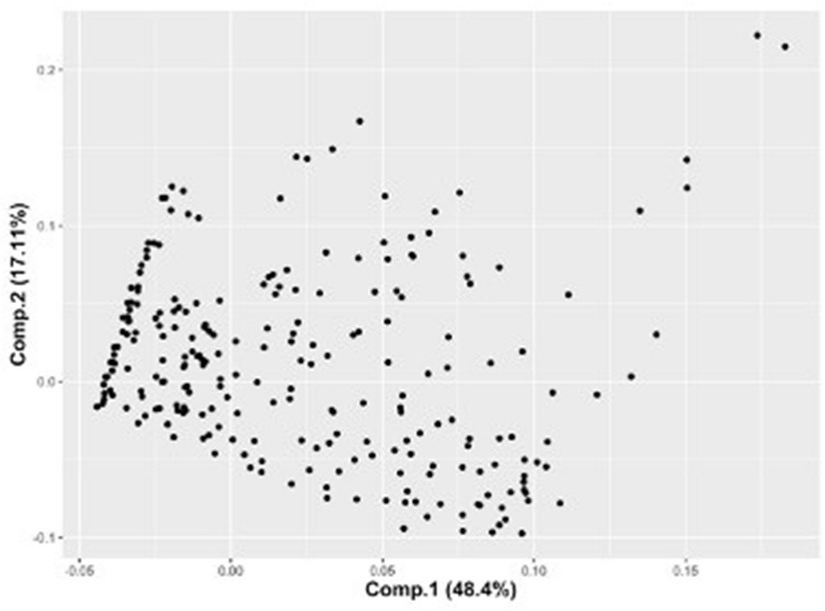
Score plot of dogs on the first (PC 1) and second (PC 2) principal components, with the increase of thunderstorms.

**Figure 4:**
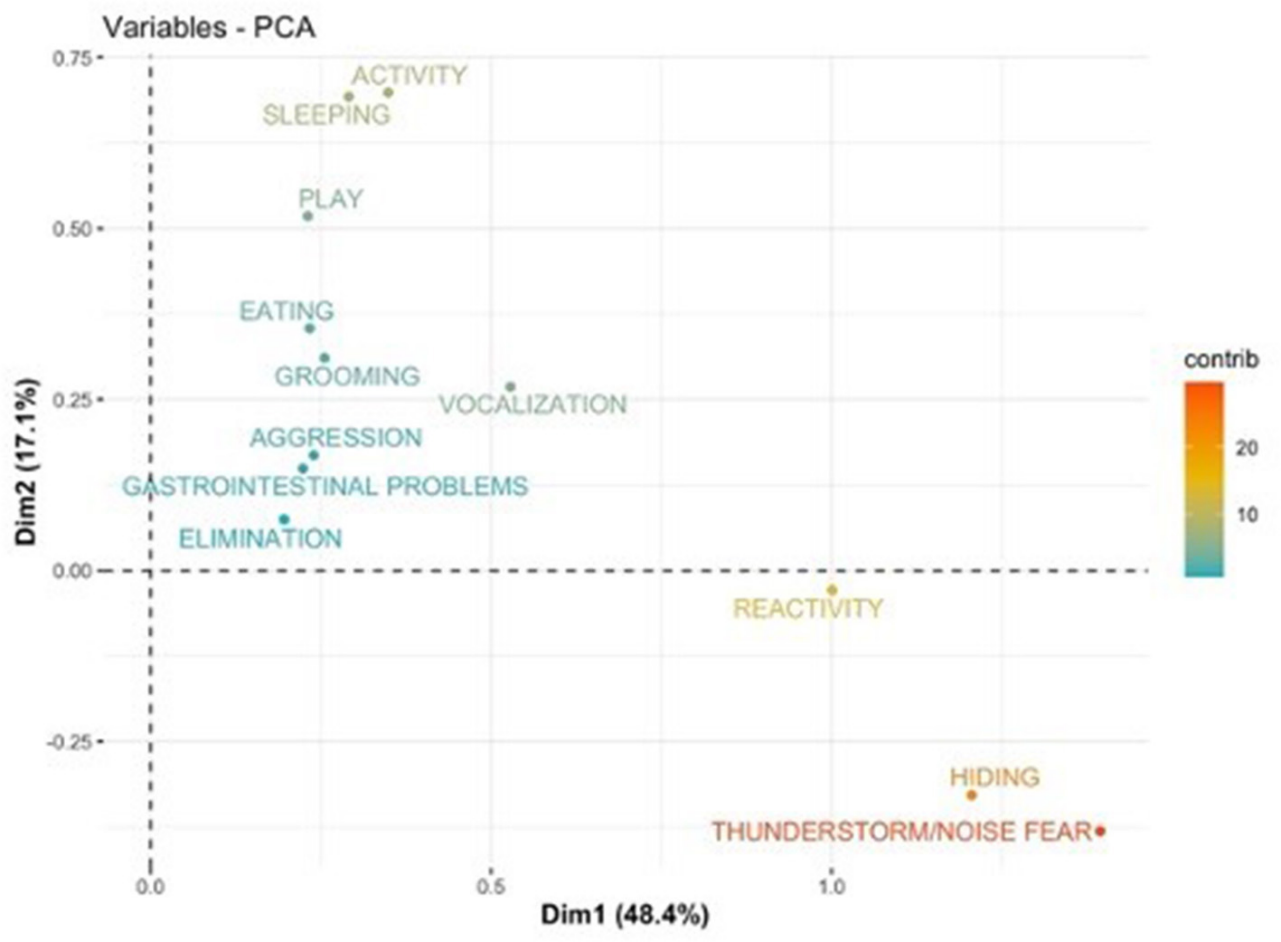
Loadings plot of the behavioral variables of dogs on the first (PC 1) and second (PC 2) principal components with the increase of thunderstorms. The contribution of the variables is colored depending on their importance.

**Figure 5:**
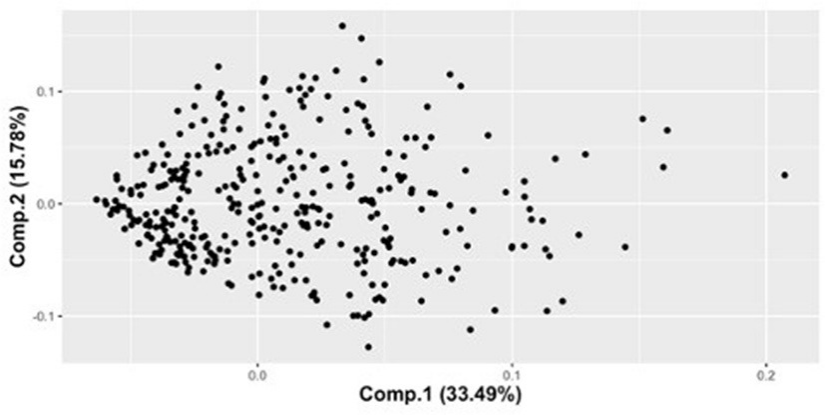
Score plot of cats on the first (PC 1) and second (PC 2) principal components, with the increase in temperature.

**Figure 6:**
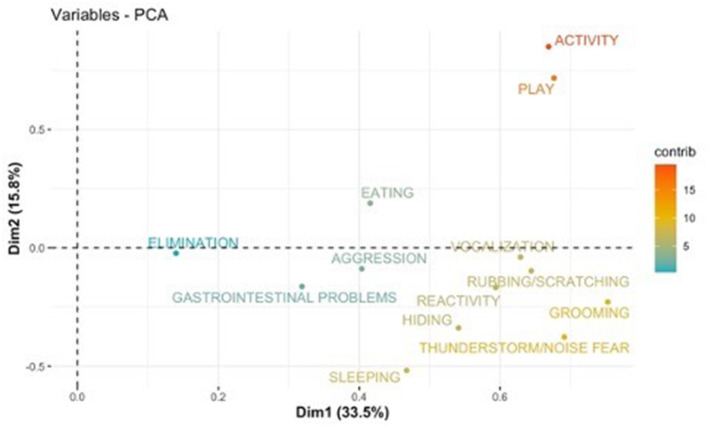
Loadings plot of the behavioral variables of cats on the first (PC 1) and second (PC 2) principal components with the increase in temperature. The contribution of the variables is colored depending on their importance.

**Figure 7:**
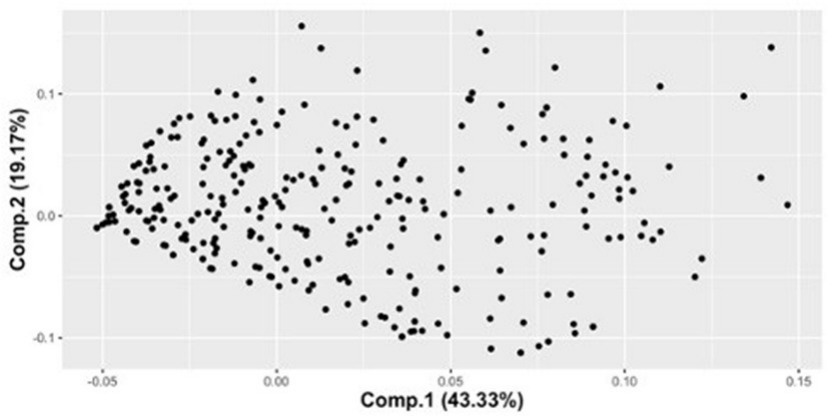
Score plot of cats on the first (PC 1) and second (PC 2) principal components, with the increase of thunderstorms.

**Figure 8:**
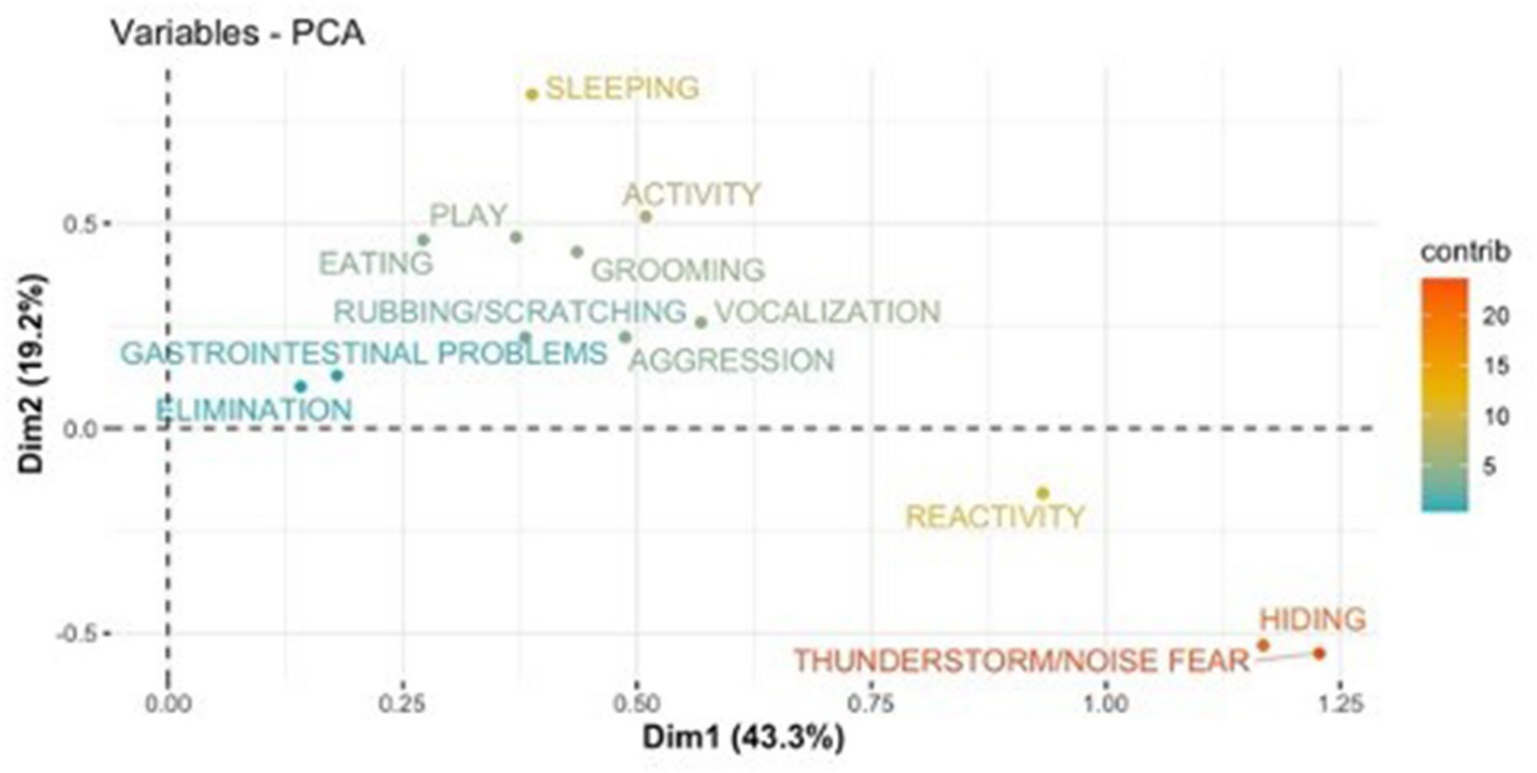
Loadings plot of the behavioral variables of cats on the first (PC 1) and second (PC 2) principal components with the increase of thunderstorms. The contribution of the variables is colored depending on their importance.

For both cats and dogs, the PCA analysis showed homogeneity of the sample: we did not find any clustering based on individuals' sex or age. Individuals with a similar profile are grouped together in the graph of individuals (cats or dogs) (Figures 1, 3, 5, 7). In the graph of variables (Figures 2, 4, 6, 8), positively correlated variables point to the same side of the plot, while negatively correlated variables point to opposite sides of the graph. The contribution of the variables is colored depending on their importance. With the increase in temperature, dogs were more differentiated by behaviors such as play and activity, thunderstorm and noise fear (Figure 2), while cats were more differentiated by behaviors such as play and activity (Figure 6). With the increase in thunderstorms, both dogs and cats were more differentiated by behaviors such as thunderstorm and noise fear, and hiding (Figures 4, 8).

## Discussion

Veterinary and medical public health are threatened by global climate change, which is considered a One Health crisis (12, 13). Weather events can directly affect dogs' and cats' behavior, since their organism implements complex physiological mechanisms to restore body homeostasis. Moreover, owners could be induced to change their management routine based on the weather conditions, and this could be a factor that indirectly influences pets' behavior. It is known that changes in daily routines create a condition of distress that modifies the main behavioral patterns (18) and worsen pre-existing pathological conditions, such as anxiety, fear and loud noises or thunderstorm phobia. Behavioral problems such as urinary marking (21, 22), separation anxiety (23), and cognitive dysfunction in elderly subjects (24) may also be exacerbated. In literature, limited attention has been given to the environmental factors that may contribute to canine and feline behavior change.

Our sample resulted to be very homogeneous in terms of both owners and pets. Our results showed that owners' and pets' age did not affect the behaviors considered in the questionnaire. As confirmed by the PCA analysis, little difference was found comparing dogs and cats, which were equally distributed between sex and age. Most owners followed a specific daily routine, even if the number and duration of outside activities were adapted to the outside temperature in extreme heat or cold periods. Heat-related illness is a potentially fatal condition inflicted on pets, bound to be more frequent as global temperatures rise. Older animals are at greater risk of developing a heat-related illness, even from sitting outside in hot weather. Extreme heat events are known to typically affect socially vulnerable patients, such as those with advanced age or chronic medical conditions, which may be confined indoors and be less resilient to natural hazards such as heatwaves (7, 28–30). Older dogs are more likely to suffer from underlying health conditions that impact thermoregulation, such as metabolic dysfunction or heart disease (31), which could increase the likelihood of environmental illnesses (32). Global warming will lead to the need to expand cooling strategies, which will inevitably include our canine and feline companions, as they can suffer fatal consequences when we fail to keep them safe (7, 27).

Our study showed that many behaviors in both species were equally modified by environmental temperature. Play and activity increased with cold weather and decreased with heat, and sleep increased with drops in temperature and with hot weather. Different factors appear to influence sleep in dogs, including diet and frequency of feeding (33), changes in housing conditions (34), changes in working routine (35), and activity levels during the day (36), but to our knowledge, no studies reported a relation of sleep and activity level to environmental temperature. Our results are possibly related to the direct influence of temperature on behavior but also to the consequence of the temperature-induced changes in daily routine.

The increase in activity in correspondence with the thermic drop was more significant in whole males; this result may be related to testosterone, which exerts a positive action on energy metabolism (37) and it could also relate to the difference in activity level between males and females and between gonadectomized and no-gonadectomized subjects (38–41).

We found a statistically significant increase in Sheepdogs and Cattle dogs (group 1) playing behavior in the presence of thermic drop: this result could be related to breed activity needs.

A lower propensity to play in high ambient temperatures could be considered a factor of rapid environmental adaptation since Hall et al. (7) found that exercise was the most common trigger of heat-related illness in dogs. Heatstroke caused by exercise was just as likely to kill as heatstroke from a hot car. Respiratory diseases, such as brachycephalic obstructive airway disorder (BOAS), have been shown to accelerate the increase in body temperature during exercise (42), and brachycephalic dogs have intrinsically greater odds of developing heat-related illness compared to dogs with longer muzzle (27). Heat regulation problems are reported to affect around a third of brachycephalic dogs (42) and obesity has been reported as a significant risk factor for death in dogs presenting with heat-related illness (44).

Our survey evidenced that feeding behavior was also affected by weather events, with an increase of feed intake during the cold season and a decrease in the heat: this type of feed control is part of the body temperature and metabolism homeostasis system (21). In cats, an increase in grooming behavior was observed in correspondence with temperature increases, probably due to the evaporative cooling losses through the skin and the hair licking (43, 45).

Weather events did not affect aggressive and house soiling behaviors in both dogs and cats.

Weather events, including wild thunderstorms, torrential rains influenced our pets' behavior. In correspondence with heavy thunderstorms or high-intensity incessant rains, increased nervousness and reactivity, fearful behaviors, tendency to hide, and vocalizations were observed. When an animal lives in a stressful situation, physiological changes occur, to prepare the animals' response to the perceived danger. From a behavioral viewpoint, the most frequently observed signs of fear are avoidance, immobility, and flight (46–48). Behavioral signs of fear may include increased vigilance, reactivity, and motor activity (pacing). There may also be excessive demands for human attention and reassurance in sociable individuals (cats or dogs). Equally, a fearful animal may show behavioral inhibition, shyness, avoidance, reduction of locomotor activity (freezing), hiding, and running away (49, 50). Stress associated with fear and anxiety can have negative impacts on health, welfare, behavior, and lifespan (49, 50), depending on both the nature of the stressor (intensity, duration, persistence, etc.) and the coping skills of the individual (51–53).

Our results evidenced that fear behaviors and vocalizations during thunderstorms were significantly less present in dogs adopted from breeders and higher present in cats older than 1 year of age and adopted from rescues. Behavioral responses to noises have a relatively high prevalence in the owned dog and cat population and are related to early sensitive periods, suggesting that early experience is an essential factor in the development of fear responses (54–56). Rescues animals therefore could have higher risk to develop high sensitivity to noise fear.

The result of this study represents a step toward the improvement of owners' awareness on management tools that can limit their animal exposure to sudden weather events. Without appropriate mitigation strategies, as extreme weather events continues to increase, also the frequency of heatwave and heavy thunderstorm events and the number of dogs and cats experiencing environmental stressors will likely to increase.

This study highlights canine and feline behavioral modification related to weather events in Italy. The main limitation of this study relates to the use of a questionnaire and the use of self-reported data. Our data refer to owners' perceptions, who detect the behaviors of their pet in the daily routine and in the presence of intense atmospheric events. Since the behaviors are not directly observed, there is a level of uncertainty in the accuracy of the results. Moreover, survey participants were recruited *via* social media and veterinarians, meaning the study likely selected for a demographic of dog and cat owners more actively engaged with their pet's health.

Understanding how dogs and cats modify their behaviors based on weather events can help to refine prevention strategies through owner education and societal changes (57). Domestic dogs and cats often share their owners' home and leisure activities, including walking, running, and other sports (55). Dogs increasingly accompany their owners to the workplace (58) and are often included in travel and holiday plans. No other species more intimately intertwines with the human lifestyle, meaning dogs potentially face similar levels of both environmental and exertional heat exposure to humans. How dogs are transported, housed, and managed will also influence heat-related illness risk. As the frequency of extreme weather events such as heat waves or intense thunderstorms is increasing, society needs to prepare strategies to mitigate their effects (59) to protect both human and canine health (44).

## Data availability statement

The raw data supporting the conclusions of this article will be made available by the authors, without undue reservation.

## Ethics statement

The studies involving human participants were reviewed and approved by Ethics Committee (reference number 5021, date 18/05/2021) University of Milan, Italy. The patients/participants provided their written informed consent to participate in this study. The animal study was reviewed and approved by Ethics Committee (reference number 5021, date 18/05/2021) University of Milan, Italy. Written informed consent was obtained from the owners for the participation of their animals in this study.

## Author contributions

Conceptualization, methodology, supervision, project administration, funding acquisition, and investigation: CP and SC. Data curation: SC, AL, and GM. Formal analysis: GM. Writing—original draft preparation: CP. Writing—review and editing: CP, SC, AL, SM, and GM. All authors have read and agreed to the published version of the manuscript.

## Funding

The authors declare that this study received funding from MSD Animal Health Srl. The funder was not involved in the study design, collection, analysis, interpretation of data, the writing of this article, or the decision to submit it for publication.

## Conflict of interest

The authors declare that the research was conducted in the absence of any commercial or financial relationships that could be construed as a potential conflict of interest.

## Publisher's note

All claims expressed in this article are solely those of the authors and do not necessarily represent those of their affiliated organizations, or those of the publisher, the editors and the reviewers. Any product that may be evaluated in this article, or claim that may be made by its manufacturer, is not guaranteed or endorsed by the publisher.
