# Supplementary material for: Do intense weather events influence dogs' and cats' behavior? Analysis of owner reported data in Italy
Source: Front Vet Sci. 2022 Sep 2;9:973574. doi: 10.3389/fvets.2022.973574 (PMC9480616; doi:10.3389/fvets.2022.973574)
Supplement: Supplementary file 1 [file Data_Sheet_1.PDF]

Supplementary materials S1: list of the climate-relevant questions included in the questionnaire

DOG QUESTIONNAIRE

1. **Demographic questions about the participant**

   1 Gender
   2 Age
   3 Household composition (number of people living together)
   4 Presence of children in the household
   5 Where do you live?
   6 Geographical characteristics of your living area: urban and suburban or rural area
   7 Type of residence: apartment, house, cottage, with or without a garden
   8 Are there in your house other pets?
   9 In your opinion, how much weather variability affects the behavior of your pet?
   10 How much weather events modify your management routine?

2. **Demographic questions about the dog**

   1. Breed
   2. Age
   3. Sex
   4. Neutering status
   5. Age at the time of adoption
   6. Source of adoption

3. **Questions related to housing, dog management, and behavior**

   1. How often is your dog taken out for a walk?
   2. How long does an average walk last?
   3. Where does the dog sleep?
   4. Do you follow a daily routine in managing your dog?
   5. How would you describe your dog's sleep?
   6. How would you describe your dog's appetite?
   7. How do you think your dog's activity level is?
   8. How do you think your dog's playing behavior is?
   9. Has your dog ever shown aggressive behavior (biting / growling / scratching)?
   10. Has your dog ever littered the house?
   11. How would you describe your dog’s grooming behavior?
   12. Is your dog very reactive, nervous, or does she/he react to every slightest stimulus?
   13. How would you describe your dog's vocalization?
   14. Is your dog fearful of loud noises (e.g. fireworks, gunshots, thunderstorms)?

4. **Questions related to weather events**
   1. During the change of seasons, when the temperature rises, how much do the aforementioned behavior (section 3) change?
   2. When the season changes, when the temperature decreases, how much the aforementioned behavior (section 3) changes?
   3. When thunderstorms with heavy rainfall occur, how intensely do the behaviors listed in paragraph 3 increase?
   4. When thunderstorms with heavy rainfall occur, how intensely do the behaviors listed in paragraph 3 decrease?

CAT QUESTIONNAIRE

1. **Demographic questions about the participant**

   1. Gender
   2. Age
   3. Household composition (number of people living together)
   4. Presence of children in the household
   5. Where do you live?
   6. Geographical characteristics of your living area: urban and suburban or rural area
   7. Type of residence: apartment, house, cottage, with or without a garden
   8. Are there in your house other pets?
   9. In your opinion, how much weather variability affects the behavior of your pet?
   10. How much weather events modify your management routine?

2. **Demographic questions about the cat**

   1. Breed
   2. Age
   3. Sex
   4. Neutering status
   5. Age at the time of adoption
   6. Source of adoption

3. **Questions related to housing, cat management, and behavior**

   1. Does the cat have access to the outside of the house?
   2. Where does the cat sleep?
   3. Do you follow a daily routine in managing your cat?
   4. How would you describe your cat's sleep?
   5. How would you describe your cat's appetite?
   6. How do you think your cat's activity level is?
   7. How do you think your cat's playing behavior is?
   8. Has your cat ever shown aggressive behavior (biting / growling / scratching)?
   9. Has your cat ever littered the house?
   10. How would you describe your cat’s grooming behavior?
   11. Is your cat very reactive, nervous, or does she/he react to every slightest stimulus?
   12. How would you describe your cat's vocalization?
   13. Is your cat fearful of loud noises (e.g. fireworks, gunshots, thunderstorms)?

4. **Questions related to weather events**
   1. During the change of seasons, when the temperature rises, how much do the aforementioned behavior (section 3) change?
   2. During the change of seasons, when the temperature decreases, how much do the aforementioned behavior (section 3) change?
   3. When thunderstorms with heavy rainfall occur, how intensely do the behaviors listed in paragraph 3 increase?
   4. When thunderstorms with heavy rainfall occur, how intensely do the behaviors listed in paragraph 3 decrease?
