# Supplementary material for: Do intense weather events influence dogs' and cats' behavior? Analysis of owner reported data in Italy
Source: Front Vet Sci. 2022 Sep 2;9:973574. doi: 10.3389/fvets.2022.973574 (PMC9480616; doi:10.3389/fvets.2022.973574)
Supplement: Supplementary file 2 [file Data_Sheet_2.PDF]

Supplementary materials S2: answer type for each questionnaire question

DOG QUESTIONNAIRE

During the change of seasons, when the **temperature rises**, do you observe:					
	Never (1)	Rarely **(2)**	Sometimes **(3)**	Very frequently **(4)**	Always **(5)**
A decrease in walks frequency					
An increase in walks frequency					
A decrease of the walk's duration					
An increase of the walk's duration					
A decrease of dog's appetite					
An increase of dog's appetite					
A decrease in dog's activity level					
An increase in dog's activity level					
A decrease in dog's playing behavior					
An increase in dog's playing behavior					
A decrease in dog's aggressive behavior					
An increase in dog's aggressive behavior					
A decrease in house littering behavior					
An increase in house littering behavior					
A decrease in dog's grooming behavior					
An increase in dog's grooming behavior					
A decrease in dog's reactivity level					
An increase in dog's reactivity level					
A decrease in dog's vocalization					
An increase in dog's vocalization					
A decrease in dog's fear of loud and sudden noises					
An increase in dog's fear of loud and sudden noises					
Changes in daily managing routine	Open question				
Changes in place where the dog sleeps	Open question				
Changes in dog's sleep	Open question				

During the change of seasons, when the **temperature decreases**, do you observe:					
	Never (1)	Rarely **(2)**	Sometimes **(3)**	Very frequently **(4)**	Always **(5)**
A decrease in walks frequency					
An increase in walks frequency					

A decrease of the walk’s duration					
An increase of the walk’s duration					
A decrease of dog's appetite					
An increase of dog's appetite					
A decrease in dog's activity level					
An increase in dog's activity level					
A decrease in dog's playing behavior					
An increase in dog's playing behavior					
A decrease in dog's aggressive behavior					
An increase in dog's aggressive behavior					
A decrease in house littering behavior					
An increase in house littering behavior					
A decrease in dog's grooming behavior					
An increase in dog's grooming behavior					
A decrease in dog's reactivity level					
An increase in dog's reactivity level					
A decrease in dog's vocalization					
An increase in dog's vocalization					
A decrease in dog's fear of loud and sudden noises					
An increase in dog's fear of loud and sudden noises					
Changes in daily managing routine	Open question				
Changes in place where the dog sleeps	Open question				
Changes in dog's sleep	Open question				

During **thunderstorms with heavy rainfall**, do you observe:					
	Never (1)	Rarely **(2)**	Sometimes **(3)**	Very frequently **(4)**	Always **(5)**
A decrease in walks frequency					
An increase in walks frequency					
A decrease of the walk’s duration					
An increase of the walk’s duration					
A decrease of dog's appetite					
An increase of dog's appetite					
A decrease in dog's activity level					
An increase in dog's activity level					
A decrease in dog's playing behavior					
An increase in dog's playing behavior					

A decrease in dog's aggressive behavior					
An increase in dog's aggressive behavior					
A decrease in house littering behavior					
An increase in house littering behavior					
A decrease in dog's grooming behavior					
An increase in dog's grooming behavior					
A decrease in dog's reactivity level					
An increase in dog's reactivity level					
A decrease in dog's vocalization					
An increase in dog's vocalization					
A decrease in dog's fear of loud and sudden noises					
An increase in dog's fear of loud and sudden noises					
Changes in daily managing routine	Open question				
Changes in place where the dog sleeps	Open question				
Changes in dog's sleep	Open question				

CAT QUESTIONNAIRE

During the change of seasons, when the **temperature rises**, do you observe:					
	Never (1)	Rarely **(2)**	Sometimes **(3)**	Very frequently **(4)**	Always **(5)**
A decrease in cat’s access to the outside of the house					
An increase in cat’s access to the outside of the house					
A decrease of cat's appetite					
An increase of cat's appetite					
A decrease in cat's activity level					
An increase in cat's activity level					
A decrease in cat's playing behavior					
An increase in cat's playing behavior					
A decrease in cat's aggressive behavior					
An increase in cat's aggressive behavior					
A decrease in house littering behavior					
An increase in house littering behavior					
A decrease in cat's grooming behavior					
An increase in cat's grooming behavior					
A decrease in cat's reactivity level					

An increase in cat's reactivity level					
A decrease in cat's vocalization					
An increase in cat's vocalization					
A decrease in cat's fear of loud and sudden noises					
An increase in cat's fear of loud and sudden noises					
Changes in daily managing routine	Open question				
Changes in place where the cat sleeps	Open question				
Changes in cat's sleep	Open question				

During the change of seasons, when the **temperature decreases**, do you observe:					
	Never (1)	Rarely **(2)**	Sometimes **(3)**	Very frequently **(4)**	Always **(5)**
A decrease in cat’s access to the outside of the house					
An increase in cat’s access to the outside of the house					
A decrease of cat's appetite					
An increase of cat's appetite					
A decrease in cat's activity level					
An increase in cat's activity level					
A decrease in cat's playing behavior					
An increase in cat's playing behavior					
A decrease in cat's aggressive behavior					
An increase in cat's aggressive behavior					
A decrease in house littering behavior					
An increase in house littering behavior					
A decrease in cat's grooming behavior					
An increase in cat's grooming behavior					
A decrease in cat's reactivity level					
An increase in cat's reactivity level					
A decrease in cat's vocalization					
An increase in cat's vocalization					
A decrease in cat's fear of loud and sudden noises					
An increase in cat's fear of loud and sudden noises					
Changes in daily managing routine	Open question				
Changes in place where the cat sleeps	Open question				
Changes in cat's sleep	Open question				

During **thunderstorms with heavy rainfall**, do you observe:					
	Never (1)	Rarely **(2)**	Sometimes **(3)**	Very frequently **(4)**	Always **(5)**
A decrease in cat's access to the outside of the house					
An increase in cat's access to the outside of the house					
A decrease of cat's appetite					
An increase of cat's appetite					
A decrease in cat's activity level					
An increase in cat's activity level					
A decrease in cat's playing behavior					
An increase in cat's playing behavior					
A decrease in cat's aggressive behavior					
An increase in cat's aggressive behavior					
A decrease in house littering behavior					
An increase in house littering behavior					
A decrease in cat's grooming behavior					
An increase in cat's grooming behavior					
A decrease in cat's reactivity level					
An increase in cat's reactivity level					
A decrease in cat's vocalization					
An increase in cat's vocalization					
A decrease in cat's fear of loud and sudden noises					
An increase in cat's fear of loud and sudden noises					
Changes in daily managing routine	Open question				
Changes in place where the cat sleeps	Open question				
Changes in cat's sleep	Open question				
